# Personalized Federated Actor–Critic Learning for Joint Cost–Comfort Optimization in Energy Communities

**DOI:** 10.3390/s26102958

**Published:** 2026-05-08

**Authors:** Sotirios Spantideas, Anastasios Giannopoulos

**Affiliations:** 1 Department of Electrical and Electronics Engineering, School of Engineering, University of West Attica, 12244 Athens, Greece; 2 School of Electrical and Computer Engineering (ECE), National Technical University of Athens, 15780 Athens, Greece; angianno@uoa.gr

**Keywords:** energy management system (EMS), energy storage systems (ESSs), heating, ventilation, and air conditioning (HVAC) systems, personalized federated learning with using Moreau envelopes (pFedMe), home automation, smart homes, deep reinforcement learning (DRL), energy community

## Abstract

Home energy management systems (HEMS) aim to provide intelligent control of the thermal comfort inside smart buildings with the minimum energy cost, while satisfying the energy consumption requests and increasing the use of energy from renewable sources. The capabilities of these intelligent HEMS agents are restricted due to the personalized observability of the environment, resulting in limited knowledge gathering and potentially sub-optimal decisions. Furthermore, several buildings have recently been organized into small energy communities, with the ultimate goal of sharing intelligence between agents in federated learning schemes.In this context, we propose a personalized federated deep reinforcement learning method using Moreau envelopes (pFedMe) for joint energy cost and household comfort optimization in energy communities that consist of multiple smart homes. Specifically, a Twin-Delayed Deep Deterministic Policy Gradient (TD3) actor–critic model is introduced, dynamically observing the state of the smart home environment and suggesting control actions on the operation of the Energy Storage System and on the regulation of the indoor temperature. The TD3 actor–critic model leads to improved policy performance in the continuous control of these systems, mitigating the overestimation bias and improving the training stability of the intelligent agents. The efficiency of the proposed method is verified via simulations based on real data, achieving a beneficial trade-off between the energy cost and the thermal comfort compared to FedAvg and Fedprox baselines. The results show that the proposed pFedMe framework consistently outperforms FedAvg and FedProx in both convergence speed and overall reward, achieving an energy cost reduction of approximately 10% compared to the other schemes, while exhibiting marginal thermal comfort behavior.

## 1. Introduction

Residential and commercial buildings are considered key contributors to worldwide energy consumption, and several technical enablers have emerged during the last few years to limit their energy footprint and provide sustainable energy management frameworks [1,2]. These frameworks aim to deliver multi-optimization solutions, ranging from thermal comfort inside a household to cost minimization related to the energy consumption and increase in the utilization of energy gathered from renewable sources [3,4,5]. In this context, energy management systems (EMS) use monitoring data generated by the building and the environment, for instance, from Internet of Things (IoT) devices, sensors, or cameras, to suggest control actions towards a fully automated solution. Moreover, they also leverage technical solutions such as Energy Storage Systems (ESS) to store the excess energy and IoT–edge–cloud continuum concepts to host the data processing and computing operations. Finally, the EMS can be assisted by Artificial Intelligence (AI) and Machine Learning (ML) models to incorporate cognitive agents that can interact with the building environment and provide long-term benefits towards optimization objectives, especially those relating to energy footprint minimization and the sustainability of smart buildings [6,7,8,9].

Although these smart residential and commercial buildings include monitoring capabilities, the design of an efficient EMS is extremely challenging, since several environmental, energy market and demand-driven parameters are involved, which exhibit combined dependencies and significant temporal variations [10,11]. To this end, the EMS must take into account periodical and seasonal variations in some parameters (for instance, the ambient temperature and the output of renewable energy generators), as well as parameters that might follow specific temporal patterns (the energy demands and loads of residential hosts that are typically increased during afternoon hours) and variables that cannot be estimated (e.g., the fluctuation in the electricity price in the energy market). Moreover, several environmental parameters are correlated, such as the use of Heating, Ventilation and Air Conditioning (HVAC) systems with ambient temperature (minimal use of HVAC is expected in cases that the outdoor temperature is in the range of 20–25 °C) or even the energy demands of the household with the electricity price, since an abrupt increase in the energy cost may prevent residents from requesting heavy consumption loads [12,13,14]. Although the inclusion of an ESS system that can act as an energy buffer is certainly a technical enabler for overcoming the latter scenario, it hinders the decision-making process imposed by the management system, since current choices of EMS impact future smart building states, requiring the configuration of an optimal EMS policy [15,16].

In this context, an intelligent EMS in a smart building must consider all the aforementioned parameters in the decision-making process of optimizing the overall energy consumption, which includes loads of inelastic demand (e.g., lights, television), controllable loads (such as the HVAC system) and adjustable loads (the operation of a washing machine can be scheduled according to the household’s needs). The key contributor to the energy consumption of a smart building is the HVAC system (up to 40% of the energy cost is linked to HVAC operation), regulating the indoor temperature between desirable limits to maximize the comfort of the residents through a feedback loop [6,17,18]. Moreover, the number of electric vehicles (EV) that are charged either autonomously or through smart buildings has been significantly increasing during the last few years, impacting grid operations and presenting a huge concern for Electric Distribution System Operators (DSOs) [19]. The use of Vehicle to Grid (V2G) bidirectional chargers also complicates the management process, since EVs can work both as loads and ESSs. The key strategy of DSOs is to balance the local energy demand requested by the loads with the available energy produced by renewable sources in close proximity to the demand, considering smart buildings and EVs as prosumers at the edge that can dynamically act as consumers and producers, exchanging energy through the local electric network [20,21,22].

In principle, DSOs tackle this problem on three different levels: first of all, at the edge (EMS, EV chargers) with intelligent algorithms that optimize energy consumption and production directly at the origin, also adjusting the data exchanged between the entities with the grid and the upper layers [23,24,25,26]. Furthermore, an additional hierarchical layer is the fog, where an energy community or an aggregator entity named Community Orchestrator has been introduced to manage the energy exchange between several prosumers (smart buildings and EVs), aiming to achieve an inter-community energy balance and also coordinate in an intra-community manner in case of energy surplus or deficit [27,28,29]. Finally, the cloud level is a centralized controlling system that regulates the energy management of multiple communities through the Community Orchestrators. Focusing on the edge and fog layers of the distributed architecture, optimization algorithms based on AI/ML have been developed, such as reinforcement learning (RL) models, where an intelligent agent can control energy-related operations of some loads of EMS and EV systems. These intelligent agents also have a cognitive awareness of the energy market, since interaction with the grid might be imperative in cases of energy deficit. It is worth noting that in the case that communication with hierarchically higher entities fails, the intelligent RL models deployed at the edge are expected to manage the local needs autonomously [30,31].

In this context, these household-level or EV-level intelligent agents relying on RL or Deep RL (DRL) models are individually trained offline, since they require interaction with a simulated environment on a trial-and-error basis [6,7]. Then, the pre-trained models can be used for online inference to provide intelligent control of the household parameters, i.e., the operation or scheduling of the loads and interaction with the energy community and the power grid. However, these models in principle exhibit generalizability issues, since they are trained based on a local dataset that reflects the local environmental conditions encountered by the agent (e.g., ambient temperature) and the resident-specific patterns for energy consumption, as well as the temporal-specific electricity price fluctuations [32,33,34]. To overcome these challenges, the DRL models can be trained in a federated configuration, where, periodically, the model weights are shared between the participants (households and EVs) during the training process, disseminating the gathered knowledge among multiple participants and converting the local observability of each intelligent agent to a more generalized perception [35,36].

## 2. Related Work and Contributions

Regarding the energy management optimization problem of an individual building, several methods have been developed in the literature. Among others, the DRL-based methods are considered dominant for solving non-convex problems with conflicting optimization objectives in complex environments [37]. As mentioned, the DRL agents interact with the environment during their training, receiving feedback based on their actions and storing their experience in a replay memory [6,38]. For instance, the authors in [17] describe the modeling of the environment and the definition of a DRL model that controls the heating in smart buildings in order to jointly reduce the energy cost and maintain a comfortable temperature range. Specifically, they use Deep Q-network (DQN) agents to the demonstrate the efficiency of the DRL with real data for the ambient temperature. Furthermore, Ref. [39] presents OCTOPUS, an HEMS system based on DRL that performs the intelligent scheduling of multiple electrical loads inside the smart building, including the HVAC system, the lighting, the blinds and the windows. The authors develop the DRL agents utilizing a branching dueling Q-network and showcase its efficacy in controlling the subsystems of the smart building environment. Similarly, the authors of [40] take into consideration the fluctuating electricity price of the market in the modeling process to configure the price of the individual energy surplus through a Deep Deterministic Policy Gradient (DDPG) agent that is used as a DRL method to provide continuous suggestions across the control actions. Finally, the authors in [41] propose a generalized actor–critic learning (GACL) optimal control method, aiming to jointly optimize the energy management of a smart home and minimize the relevant cost by introducing three iteration processes in the GACL algorithm, namely, the global iteration, the local iteration, and the interior iteration.

Apart from training individually cognitive agents, multiple research studies that deal with collaborative intelligent schemes can be found in the literature. Specifically, the authors of [32] propose a federated reinforcement learning (FRL) approach for HEMS in homes with appliances/loads, a solar photovoltaic system as a renewable energy sources, and an ESS. The distributed HEMS agents are trained with local energy consumption data, uploading their local model to a global server (GS) that aggregates the local models into a global model, redistributing it back to the local HEMS. Moreover, the authors in [42] present a hierarchical framework for enabling collaborative intelligence between multiple smart homes using federated deep reinforcement learning (FDRL). In the context of the proposed framework, the intelligent HEMS agents that manage the smart building, consisting of ESS, loads and renewables, share their gained experience as model hyperparameters through a federation layer with the optimization objective of reducing costs and CO2 emissions. In addition, Ref. [43] introduces a privacy-preserving FRL method that uses a shared ESS for multiple smart buildings, describing a hierarchical architecture based on a GS and local HEMS clients. To this end, the authors extensively describe how the FL framework is deployed, i.e., how the GS aggregates the local trained neural networks for energy consumption models and broadcast the global model, as well as training the shared ESS system to exchange energy (charging and discharging operations) between the utility grid and smart buildings. Finally, the authors of [44] propose a personalized energy management approach for each smart home that participates in the FL process, based on the method of personalized federated deep reinforcement learning (PFDRL). In particular, the proposed approach includes a global energy management strategy using FDRL, as well as a personalized fine-tuning scheme based on the divergence between the personalized strategy of each individual agent and the global strategy.

Recent studies have also explored advanced actor–critic architectures and their integration with communication-constrained or distributed environments. In [45], the authors propose an MSRA-TD3 framework for throughput maximization under Age of Information (AoI) constraints in energy-harvesting D2D-enabled cellular networks, demonstrating the stability and improved convergence of TD3 in stochastic and resource-constrained settings. Although this work highlights the advantages of TD3 over traditional actor–critic methods, it focuses on communication networks rather than energy management in smart buildings. Similarly, TD3-based approaches have been employed in residential microgrid energy management, achieving improved convergence and enhanced trade-offs between energy cost and user comfort [46]. Other works have investigated TD3 for power scheduling in distributed renewable energy systems and energy management in hybrid electric vehicles, demonstrating its robustness in handling continuous control tasks under uncertainty [47,48]. Moreover, recent extensions combine TD3 with optimization techniques, such as particle swarm optimization, to further enhance performance in energy harvesting and wireless network scenarios [49].

The authors in [50] investigate multi-agent reinforcement learning for coordinated energy management across multiple buildings, but rely on centralized training mechanisms, which may raise scalability and privacy concerns. On the federated learning side, recent works such as [51,52] have introduced personalized FL frameworks that decouple global model learning from local personalization using regularization techniques, including Moreau envelopes. While these methods provide strong theoretical guarantees and improved personalization, their application to DRL-based control problems, particularly in continuous control environments such as HEMS, remains largely unexplored.

In practical energy communities, smart homes may operate under highly heterogeneous conditions, including different occupancy patterns, thermal dynamics, appliance usage profiles, and local renewable generation. This heterogeneity makes it challenging to learn a single global control policy that performs well across all participants, as traditional federated learning approaches such as FedAvg tend to average out local characteristics, leading to sub-optimal decisions for individual homes. At the same time, directly sharing raw energy consumption and behavioral data is undesirable due to privacy concerns and communication constraints. To address these challenges, we adopt a personalized federated deep reinforcement learning framework based on Moreau envelopes (pFedMe), which enables collaborative learning without exposing sensitive data while explicitly accounting for local differences. The key mechanism in pFedMe is to decouple global knowledge sharing from local model adaptation through a regularized optimization process, allowing each smart home to benefit from the collective experience of the community while maintaining a personalized control policy tailored to its own environment.This is particularly important in the considered energy communities, where the trade-off between energy cost and thermal comfort is inherently user-dependent, and thus requires individualized decision-making strategies. The proposed approach combines TD3-based continuous control with personalized federated learning for joint optimization of energy cost and indoor comfort, thereby addressing both system heterogeneity and privacy-preserving learning in smart home energy management. The key innovations of the present work include the following:

- We develop a comprehensive smart home energy management model that captures dynamic energy demand, renewable generation, electricity pricing, and indoor thermal dynamics, while incorporating an ESS for local energy balancing [7,18].
- We formulate a joint optimization problem for minimizing energy cost and maximizing indoor comfort, and solve it using a TD3 actor–critic framework for continuous control of HVAC operation and ESS scheduling.
- We extend the single-agent setting to an energy community scenario by proposing a PFDRL learning framework based on personalized FL using Moreau envelopes (pFedMe), enabling collaborative learning while preserving user-specific preferences [52].
- We validate the proposed approach using real-world data, demonstrating improved convergence and performance over FedAvg and FedProx, achieving over 10% energy cost reduction while maintaining temperature deviations within 1.5 °C.

## 3. System Model

### 3.1. Smart Home Modeling

**Figure 1:**
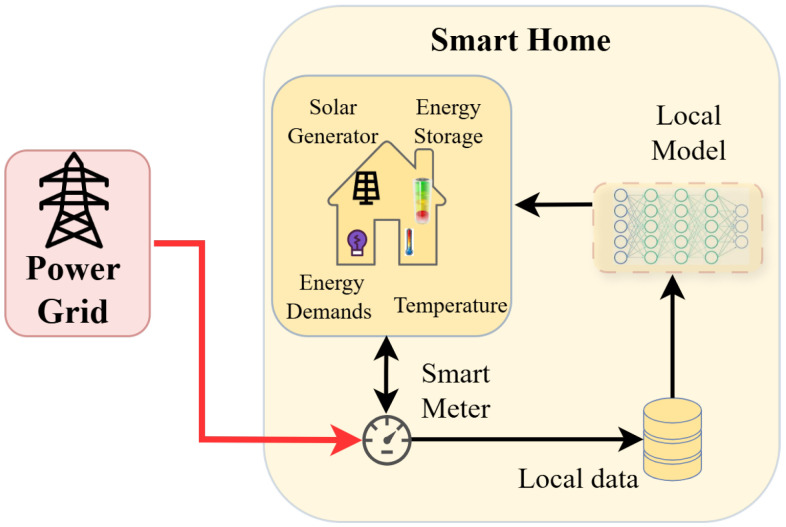
Smart home environment including the energy consumption demands, the energy generated from solar panels, the ESS, and the indoor and outdoor temperature, as well as the electricity price, which are included in the TD3 local model for training (the red arrow illustrates the power flow, whereas the black arrows the data flow).

The modeling of an individual smart home environment is shown in Figure 1. Without loss of generality, it is assumed that the energy generators included in the smart home are solar generators that are used to gather power from the sunlight energy during the day. Moreover, the smart home also exhibits varying energy demands, i.e., energy consumption requests that reflect the normal operations of the households. To this end, three types of loads are present in a smart home: (i) non-shiftable requests that require the immediate provision of energy (e.g., lights, oven operation); (ii) shiftable and non-interruptible loads (e.g., the operation of a washing machine); (iii) controllable loads (e.g., the HVAC system) that can be scheduled or regulated over a period of time. Apart from the energy generators and loads, an ESS is also present in the environment, enabling dynamic charging or discharging depending on its capacity and the current electricity price. The ESS is used as a buffer to cover the local and immediate demands by providing its stored energy. In addition, temperature sensors provide indications of the indoor temperature experienced inside the residence, as well as the outdoor (ambient) temperature.

The aforementioned data constitute the smart home environment and are conveyed to the smart meter in order to calculate the energy balance of the smart home. In case of a deficit (the local energy is not adequate for the loads of the smart home), the smart meter requests that the utility grid provide the missing energy at the current market price, which is usually a costly option. The temporal data gathered from the smart home can be stored in a local database, to be used for training a DRL model to act on the environment, i.e., regulate the input energy into the HVAC system (one of the most significant contributors to controllable loads), as well as the online operation of the ESS, i.e., draw or provide energy depending on the current market price, the energy demands of the household, the state of charge level of the storage system, etc.

In the context of mathematically formulating the optimization problem, we can denote the time-varying energy that is gathered by the solar panels as $p_{t}$ and the fluctuating energy demands of the smart home (the sum of non-shiftable, non-interruptible and controllable requests apart from the HVAC and ESS control) as $\omega_{t}$. It is worth noting that *t* is the time duration that is associated with the period of the provided measurements. Although the duration of each time slot is typically in the order of one hour, the described modeling can be applied to shorter time scales, depending on the required control granularity. Furthermore, the temporal evolution of the indoor temperature in a smart building environment can be expressed by [53,54,55]:

(1) $$T_{t+1}^{in}=\epsilon T_{t}^{in}+\left(1-\epsilon \right)\left(T_{t}^{out}-e_{t}\frac{\eta }{A}\right),$$

where $T_{t}^{out}$ is the ambient temperature that is provided by the temperature sensors; ϵ is the inertia factor, which provides a trade-off between the indoor and the outdoor temperatures; η is the coefficient that represents the performance of the HVAC system (different for cooling and heating modes); and *A* is the thermal conductivity of the smart building measured in kW/°C. The mathematical symbols that are used in the present work are summarized in Table 1.

Equation (1) indicates that the outdoor temperature, the power provided by the HVAC system $e_{t}$ (scaled by the building parameters) and its previous value affect the current indoor temperature. Although more complex equations of the indoor temperature that accurately describe the building thermal dynamics can be used to provide a more realistic representation, it is worth noting that they can be effortlessly adapted in the presented approach by replacing this equation [56]. Taking into account the capability of the HVAC system, the provided power is constrained by a maximum value $e^{max}$. The restriction is expressed by:

(2) $$0\leq e_{t}\leq e^{max},$$

where $e^{max}$ is the peak power in Watts consumed by the HVAC system during the time slot *t* (typically one hour). An additional constraint that represents the comfort experienced by the smart home residents is related to the indoor temperature $T_{t}^{in}$, which must be within specific limits:

(3) $$T^{min}\leq T_{t}^{in}\leq T^{max}.$$

To this end, the HEMS controls the HVAC power $e_{t}$ (conforming to the relevant constraint) in the current time slot *t* through Equation (1) in order to regulate the indoor temperature $T_{t+1}^{in}$ in the next time slot within the desired limits of Equation (3). Concerning the modeling of the ESS dynamics, the charge level in time slot *t* can be expressed by [7]

(4) $$C_{t+1}=C_{t}+\left(\delta_{\sigma }\sigma_{t}+\frac{\lambda_{t}}{\delta_{\lambda }}\right)\Delta t,$$

where $0<\delta_{\sigma },\delta_{\lambda }\leq 1$ are the charging and discharging coefficients, respectively, that reflect the quality of these operations. In practice, these coefficients measure the ratio of energy stored in a battery to the energy consumed/provided from/to the grid, accounting for energy losses (heat, conversion) and typically ranging from 85% to 95% for modern chargers. Moreover, the variables $\sigma_{t}$ and $\lambda_{t}$ denote the charging and discharging rates during the time slot *t*, measured in Ah or Wh. The charge level at a particular time slot is bounded within specific limits (adjusted by the capacity of the ESS) as follows:

(5) $$C^{min}\leq C_{t}\leq C^{max}.$$

The ESS charging from the grid is bounded by the following constraint:

(6) $$0\leq \sigma_{t}\leq \sigma^{max},$$

while the discharging operation to the grid follows a similar constraint, as described in the following equation:

(7) $$-\lambda^{max}\leq \lambda_{t}\leq 0.$$

In Equation (6), we have denoted the charging variable as positive-valued with respect to the charging state, whereas in Equation (7) the discharge is a negative-valued parameter, since the latter operation reduces the current charge level of the ESS. Moreover, we assume that the ESS can be used either to charge or to discharge during a time slot *t*, i.e.,

(8) $$\sigma_{t}\cdot \lambda_{t}=0.$$

Aggregating the energy consumed by the residential loads, the HVAC and the ESS (in case of charge), as well as the energy produced by the solar panels and the ESS (in case of discharge), the household-level time-varying power balance equation can be expressed by:

(9) $$p_{t}-\lambda_{t}+\gamma_{t}=\omega_{t}+\sigma_{t}+e_{t}.$$

The introduced variable $\gamma_{t}$ in Equation (9) represents the power in kW that must be drawn from the utility grid in cases where the smart home exhibits a deficit for the current time slot ($\gamma_{t}>0$), or the power that can be provided back to the grid in case of energy surplus ($\gamma_{t}<0$). It should be noted that in the former scenario, the fluctuating market price (assuming that the customer is charged according to the online market price and not with a fixed rate) for buying the missing energy, denoted as $\kappa_{t}$, is typically higher than the selling electricity price $\beta_{t}$, exhibiting a linear relation:

(10) $$\kappa_{t}=\xi \beta_{t},$$

where ξ is the scaling factor and ξ≥1. To this end, the energy balance of the smart home indicates whether power will be bought from the grid at a higher price, inherently encouraging the use of local renewable sources in smart home energy consumption. It should be noted that this modeling approach considers the grid as an infinite source that always provides the missing energy to the energy community. In addition, a linear relationship is assumed between the buying and selling energy price for simplicity; more complex equations (e.g., non-linear) can be introduced, without affecting the proposed algorithm.

### 3.2. Energy Cost Minimization Problem

Following the presented modeling process, an optimization problem is formulated for each individual smart home, i.e., the minimization of the energy cost, respecting the various constraints that are imposed from the environment and the equipment. Thus, the minimization problem involves the cost paid directly from the grid when drawing energy (either to charge the ESS or to provide energy to the HVAC in order to control the indoor temperature), as well as the indirect equipment cost of the ESS, since its continuous operation incurs a depreciation cost. Formally, the energy cost that is bought from the grid after the internal balance of the smart home is $\kappa_{t}\gamma_{t}$ (in case $\gamma_{t}>0$) and the cost related to the ESS operation is $\upsilon (\sigma_{t}+|\lambda_{t}|)$, where υ is defined as a depreciation coefficient with units $/kW. The latter term reflects the depreciation cost due to the continuous operation of the ESS system, i.e., the frequent charging or discharging operations incur a implied cost that is taken into consideration in the formulation of the energy cost minimization problem. The optimization problem can be expressed as:

(11) $$(P_{1}):\min_{e_{t},\sigma_{t},\lambda_{t}}\sum_{t=1}^{S}E\left\{\kappa_{t}\gamma_{t}+\upsilon \left(\sigma_{t}+\left|\lambda_{t}\right|\right)\right\}$$

$$\mathrm{subject}\;\mathrm{to}\;\;\;\;\begin{array}{c} \left(1\right)\text{–}\left(10\right),\;\gamma_{t}>0, \end{array}$$

where the expectation operator E is calculated for a temporal horizon of t=1,2,…S slots and the relevant time-varying environmental parameters, since their statistical distribution cannot be accurately described and can be considered random.

In principle, $\left(P_{1}\right)$ is a very complex problem with multiple challenges: (i) the thermal dynamics inside the smart home environment are, in general, very complex and cannot be accurately modeled or described; (ii) the control variables for the minimization of the energy cost $\left(e_{t},\sigma_{t},\lambda_{t}\right)$ are not independent, since the ESS system can operate in one mode (either charging or discharging), influencing the power balance of the system and, thus, the power required for the HVAC; (iii) the power balance of the building involves multiple system parameters that cannot be known beforehand. For instance, the power gathered from renewable sources and the energy demands of a residential smart home typically follow periodical temporal patterns. However, the future values of these variables can be predicted with limited accuracy. Moreover, the energy market price exhibits strong temporal fluctuations and marginal forecasting abilities; (iv) the set of control actions that are decided in a time slot $\left(e_{t},\sigma_{t},\lambda_{t}\right)$ have an impact on future control decisions of the system, potentially leading to sub-optimal solutions. For instance, charging the ESS on a day with increased market prices may be the optimal solution in cases where, in the following days, the energy demands of the household are increased, and also reduced sunlight prohibits the collection of renewable energy. To this end, the optimization problem should be solved over a finite time horizon, where the variables describing the smart home environment are statistically unstable and not for individual time instances. Finally, dynamic parameters of the environment exhibit complex inter-dependencies that cannot be easily modeled. For instance, high energy market prices can lead to reduced energy consumption and vice versa (these variables are not controlled by the model).

$\left(P_{1}\right)$ also involves conflicting optimization objectives, since the target is to minimize the energy cost, while at the same time respecting the indoor temperature constraints. The set of controlling parameters to be determined in each time slot *t* is the triplet ($e_{t}$, $\sigma_{t}$, $\lambda_{t}$) that has a direct impact on the smart home indoor environment and $\left(P_{1}\right)$ can be approximated as a decision-making Markov Decision Process (MDP) problem [7]. Specifically, most of the key smart home parameters of the t+1 time slot depend on their values in the previous time step *t* and the performed actions, e.g., the ESS level $C_{t+1}$ is calculated based only on the current level $C_{t}$ and the discharging/charging action $(\sigma_{t}$ or $\lambda_{t})$. Similarly, the indoor temperature at the next time instance t+1 depends on the indoor temperature at time *t*, the HVAC power input, the ambient temperature $T_{t}^{out}$ and the building’s characteristics. It is worth noting that some environmental parameters are not Markovian, i.e., they may follow specific patterns (e.g., increased energy consumption requests from the smart home during the afternoon) or long-range temporal correlations (e.g., renewable power generation surges during the summer months). However, $\left(P_{1}\right)$ can be still approximated as an MDP and solved by reinforcement learning algorithms [57,58].

## 4. Reinforcement Learning Model and Proposed Algorithm

This section presents the key parameters of the RL-based actor-critic model that is incorporated in the HEMS of each smart home, as shown in Figure 1, and describes the PFDRL algorithm that is applied inside the energy community.

### 4.1. Twin-Delayed Deep Deterministic Policy Gradient Model

In order to solve the optimization problem $\left(P_{1}\right)$, the intelligent HEMS agent regulates two key parameters: the power provided to the HVAC system and the power that is charged/discharged to/from the ESS. When training the RL algorithm, the TD3 agent observes the smart home parameters (environment state) in each time slot, decides upon the input power to HVAC and ESS charge/discharge (action) and then receives positive or negative feedback when the selected action is applied to the smart home (reward). To this end, the intelligent agent is trained on a trial-and-error basis, slowly learning the most beneficial actions towards the optimization objective (minimization of the energy cost) [7,54,58]. The specific DRL agent is selected since it can deal with continuous states and actions (the state and action spaces are not required to be discrete) that are described below, along with the reward function.

#### 4.1.1. Environment State

The state of the smart home that is observed by the intelligent agent, as shown in Figure 1, includes the dynamic environmental and system parameters and can be expressed as follows:

(12) $$s_{t}=\left(p_{t},\omega_{t},C_{t},T_{t}^{out},T_{t}^{in},\kappa_{t},t^{\prime }\right),$$

where the variables $p_{t},\omega_{t},C_{t},T_{t}^{out},T_{t}^{in}$ can be acknowledged to the agent through the smart meter and installed sensors, since they reflect on smart home-specific variables (energy consumption, energy generation, ESS level, ambient and indoor temperate), the current market price $\kappa_{t}$ can be retrieved online (https://data.nordpoolgroup.com/auction/day-ahead/prices?deliveryDate=latest&currency=EUR&aggregation=Hourly&deliveryAreas=AT accessed on 27 February 2026) and the variable $t^{\prime }$ denotes the time slot index in a day $t^{\prime }=mod\left(t,24\right)$.

#### 4.1.2. Action of the TD3 Agent

As aforementioned, the purpose of the TD3 agent is to adjust the power provided to the HVAC system and the power that is charged/discharged to/from the ESS. The action can be described by:

(13) $$\alpha_{t}=\left({\hat{e}}_{t},{\hat{\zeta }}_{t}\right),$$

where $0\leq {\hat{e}}_{t}\leq 1$ is the scaled HVAC input power (divided by $e^{max}$) and $-1\leq {\hat{\zeta }}_{t}\leq 1$ is the scaled variable for the ESS charging/discharging that can be computed as follows:

(14) $$\left\{\begin{array}{c} {\hat{\zeta }}_{t}=\sigma_{t}/\sigma^{max},\;\mathrm{if}\;{\hat{\zeta }}_{t}\geq 0\\ {\hat{\zeta }}_{t}=-\lambda_{t}/\lambda^{max},\;\mathrm{if}\;{\hat{\zeta }}_{t}<0. \end{array}\right.$$

To this end, the suggested TD3 actions can be unscaled and implemented in the smart home environment. The HVAC input power can be unscaled according to (13) and the single variable ${\hat{\zeta }}_{t}$ that is required to denote the charging/discharging of the ESS can be unscaled according to (14).

#### 4.1.3. Reward Function

Once the intelligent agent recommends the action vector $\alpha_{t}$, it is applied to the smart home, leading to new environment state $s_{t+1}$ in the next time slot, according to the building dynamics and the constraints described by (1)–(9). To quantify whether the selected action indeed provides the thermal comfort and cost benefits of the HEMS, a reward is calculated as:

(15) $$r_{t}\left(s_{t},\alpha_{t}\right)=-\left({\left[T_{t+1}^{in}-T^{max}\right]}^{+}+{\left[T^{min}-T_{t+1}^{in}\right]}^{-}\right)-\rho \left(\kappa_{t}\gamma_{t}+\upsilon \left(\sigma_{t}+\left|\lambda_{t}\right|\right)\right).$$

In (15), the first term reflects the experienced comfort originating from the indoor temperature, i.e., the penalty (negative reward) is zero if it is within the specified limits and equals the deviation when the indoor temperature exceeds $T^{max}$ or is below $T^{min}$. The second term represents the cost of the energy that is directly bought from the grid $\kappa_{t}\gamma_{t}$, in case that $\gamma_{t}>0$ after the power budget calculation. In addition, we penalize the energy cost according to the optimization objectives of the problem and we do not provide a positive reward in case of $\gamma_{t}<0$, i.e., the smart home exhibits energy surplus that can be sold back to the grid. Similarly, the third term of (15) is related to the ESS, since the charging/discharging operations incur a depreciation cost. Finally, ρ is a trade-off variable that balances the penalty due to the temperature deviation (typically in units of °C) and the energy cost negative reward (expressed in $).

To this end, the learning process can be tuned to be more sensitive to the indoor comfort at the expense of the energy cost or vice versa.It should be noted that additional metrics that are involved in the indoor comfort can be included in the smart home model and the training process, such as the relative humidity. The dynamics of the humidity can be modeled similarly to the indoor temperature and an additional action of the TD3 agent can be added to regulate the humidity by configuring the HVAC system [56]. It is also worth noting that the energy cost penalty term of the reward function inherently boosts the use of renewable energy that is generated locally by each smart home, eliminating the dependency from the power grid.

As is evident from the action of the TD3 agent and the reward function during the learning process, the aim of the intelligent HEMS agent is to adjust the power level provided to the HVAC system, as well as the charging or discharging power for the ESS operation in each time instance to receive the maximum reward, as expressed in (15), also called the immediate reward. Specifically, the reward is maximized (ideally zero) when the DRL agent maintains the indoor temperature within the required limits and the energy cost that is related to the HVAC and the ESS operation (depreciation cost) are both minimized. To this end, the DRL formulation completely aligns with the optimization problem under investigation and its constraints, enabling the TD3 agent to provide beneficial solutions for $\left(P_{1}\right)$. It is worth noting that the TD3 agent also inherently boosts the utilization of the generated renewable energy to cover the individual demands of the smart home, minimizing the grid energy exchange and the associated cost.

### 4.2. Individual Smart Home Algorithm

This subsection details the algorithmic process of the individual TD3 agent training. As aforementioned, the TD3 algorithm belongs to the categories of actor/critic models that deal with problems involving continuous state and action spaces. Through the continuous interaction between the actor and critic components, the policy and value function of the Td3 algorithm are updated [59]. Specifically, the role of the actor neural network is to output the action $\alpha_{t}$ solely based on the environment state $s_{t}$, providing the most beneficial decision during the inference phase of the model. On the other hand, the utilization of the twin critic networks are limited to the training phase, receiving the state vector from the smart home environment and the suggested action from the actor network. Their role is to evaluate the action recommended by the actor through an action value function $Q_{1}\left(s_{t},\alpha_{t}\right)$ and $Q_{2}\left(s_{t},\alpha_{t}\right)$, respectively. The mechanisms of clipped double Q-learning (the minimum of the target action value functions is selected) and delayed policy updates (actor is not updated in each step) are used to calculate the policy gradient and update the weights of the actor network.

During the training phase, the individual TD3 agents interact with the smart home parameters on a trial-and-error basis for multiple episodes (different instantiations of the environment), i.e., observing the state, performing actions and receiving rewards. These tuples are stored in a memory experience replay which, along with the actor and critic target networks mechanisms, are used to update the weights of the networks [60]. The algorithmic process for the training of an individual smart home TD3 agent is illustrated in Algorithm 1. The required input data are the historical datasets of renewable power generation from the solar panels $p_{t}$, the non-shiftable and non-interruptible energy demands of the household $\omega_{t}$, the time series of the outdoor ambient temperature $T_{t}^{out}$ and the buying electricity price $\kappa_{t}$, data that concern the same temporal period.

The algorithm begins by initializing the replay memory R that hosts the pool of transition tuples ($s_{t},\alpha_{t},r_{t},s_{t+1}$), the twin critic networks $Q(s_{t},\alpha_{t}|w^{Q_{1}})$, $Q(s_{t},\alpha_{t}|w^{Q_{2}})$ and the actor network $\psi (s_{t}|w^{\psi })$. In addition, the target critic and actor networks are initialized by copying the weights from the original networks $w^{Q_{1}^{\prime }}\leftarrow w^{Q_{1}}$, $w^{Q_{2}^{\prime }}\leftarrow w^{Q_{2}}$ and $w^{\psi^{\prime }}\leftarrow w^{\psi }$. The loop of the algorithm is then executed for *M* training episodes, where the environment is reset, i.e., a random time instance is selected in the dataset, representing different $p_{t},\omega_{t},T_{t}^{out},\kappa_{t}$ variables. A random ESS level $C_{t}$ and an indoor temperature $T_{t}^{in}$ are also selected to constitute the state space $s_{t}$. For each training episode, the TD3 agent interacts with the environment in within-episode time slots *N* in order to become familiarized with the time-varying dependencies of the related parameters (e.g., patterns of outdoor temperature, generation of renewable energy).To this end, the agent selects an action $\alpha_{t}$ expressed by:

(16) $$\alpha_{t}=\psi \left(s_{t}|w^{\psi }\right)+\nu ,\nu ∼N\left(0,\sigma_{n}^{2}\right).$$

According to (16), the action selected by the agent contains the exploitation term $\psi (s_{t}|w^{\psi })$, which is the vector suggested by the actor network. This term is deviated by ν, following a normal distribution with a variance of $\sigma_{n}^{2}$, to introduce exploration noise to the selected action related both to the HVAC input power and the ESS charging/discharging. The selected action $\alpha_{t}$ is applied in the environment by calculating the dependent dynamic parameters using (1), (4), and (9) and the next state $s_{t+1}$ can be acknowledged to the agent, along with the reward $r_{t}$ according to (15). This transition tuple is then stored in the replay memory R, as described in Algorithm 1.
**Algorithm 1** Training of an individual TD3 agent**Input:** 
Time series of renewable power generation, non-shiftable and non-interruptible energy demands, outdoor ambient temperature and buying electricity price.**Output:** 
Actor network $w^{\psi }$ and twin critic networks $w^{Q_{1}},w^{Q_{2}}$ of the TD3 model. 1:Random initialization of replay memory R of size *K*. 2:Random initialization of actor network $\psi (s_{t}|w^{\psi })$ and twin critic networks $Q_{1}\left(s_{t},\alpha_{t}|w^{Q_{1}}\right)$, $Q_{2}\left(s_{t},\alpha_{t}|w^{Q_{2}}\right)$. 3:Initialize target networks: 4:$w^{\psi^{\prime }}\leftarrow w^{\psi }$, $w^{Q_{1}^{\prime }}\leftarrow w^{Q_{1}}$, $w^{Q_{2}^{\prime }}\leftarrow w^{Q_{2}}$. 5:**for** ep=1,2,…,M **do** 6:   Random initialization of parameters $C_{t}$, $T_{t}^{in}$ and time instance *t*, assemble state: 7:   $s_{t}=\left(p_{t},\omega_{t},C_{t},T_{t}^{out},T_{t}^{in},\kappa_{t},t^{\prime }\right)$. 8:   **for** t=1,2,…,N **do** 9:      Select action with exploration noise:10:     $\alpha_{t}=\psi \left(s_{t}|w^{\psi }\right)+\nu$, $\nu ∼N(0,\sigma^{2})$11:     Apply action using (1), (4), (9), observe $s_{t+1}$.12:     Compute reward $r_{t}$ using (15).13:     Store transition $\left(s_{t},\alpha_{t},r_{t},s_{t+1}\right)$ in R.14:     Sample a random mini-batch of *Y* tuples $\left(s_{i},\alpha_{i},r_{i},s_{i+1}\right)$ from R, 1≤i≤Y.15:     Add clipped noise to target action:16:     ${\tilde{\alpha }}_{i+1}=\psi^{\prime }\left(s_{i+1}|w^{\psi^{\prime }}\right)+\mathrm{clip}\left(\nu ,-c,c\right)$17:     Compute target value:18:     $y_{i}=r_{i}+\theta \min_{j=1,2}Q_{j}^{\prime }\left(s_{i+1},{\tilde{\alpha }}_{i+1}\right)$19:     Update both critics by minimizing:20:     $\frac{1}{Y}\sum_{i}{\left(Q_{1}\left(s_{i},\alpha_{i}\right)-y_{i}\right)}^{2}+{\left(Q_{2}\left(s_{i},\alpha_{i}\right)-y_{i}\right)}^{2}$21:     **if** tmodd=0 **then**22:        Update actor policy:23:        $\nabla_{w^{\psi }}\frac{1}{Y}\sum_{i}Q_{1}\left(s_{i},\psi \left(s_{i}|w^{\psi }\right)\right)$24:        Soft update target networks:25:        $w^{Q_{j}^{\prime }}\leftarrow \chi w^{Q_{j}}+\left(1-\chi \right)w^{Q_{j}^{\prime }}$, j=1,226:        $w^{\psi^{\prime }}\leftarrow \chi w^{\psi }+\left(1-\chi \right)w^{\psi^{\prime }}$27:     **end if**28:   **end for**29:**end for**

Following the algorithmic process, a mini-batch of *Y* transition tuples is sampled from the replay memory and used to calculate the expected state action values. To this end, the next action is calculated by:

(17) $${\tilde{\alpha }}_{i+1}=\psi^{\prime }\left(s_{i+1}|w^{\psi^{\prime }}\right)+\mathrm{clip}\left(\nu ,-\nu_{c},\nu_{c}\right),$$

where the target actor network is used to provide the feedforward operation and the noise is clipped within the limits $\left[-\nu_{c},\nu_{c}\right]$. Then, the target Q-value can be computed for both critic networks based on the Bellman equation [59,61]:

(18) $$y_{i}=r_{i}+\theta \min_{j=1,2}Q_{j}^{\prime }\left(s_{i+1},{\tilde{\alpha }}_{i+1}\right),$$

where the target critic networks are used to compute the expected state action values and θ∈[0,1] is the discount factor that balances between the immediate and future rewards. Notably, the future rewards of the Bellman Equation (18) provide the quality of the current actions of the TD3 agent considering upcoming time instances of the complex environment. The weights of the critic networks are updated based on

(19) $$\frac{1}{Y}\sum_{i}{\left(Q_{1}\left(s_{i},\alpha_{i}\right)-y_{i}\right)}^{2}+{\left(Q_{2}\left(s_{i},\alpha_{i}\right)-y_{i}\right)}^{2}$$

and the sampled policy gradient is calculated once every policy delay time, denoted *d* for each update of the Q-networks:

(20) $$\nabla_{w^{\psi }}\frac{1}{Y}\sum_{i}Q_{1}\left(s_{i},\psi \left(s_{i}|w^{\psi }\right)\right)$$

is used to update the actor policy. Finally, the target critic and actor networks are updated by using $w^{Q_{j}^{\prime }}\leftarrow \chi w^{Q_{j}}+\left(1-\chi \right)w^{Q_{j}^{\prime }}$, j=1,2 and $w^{\psi^{\prime }}\leftarrow \chi w^{\psi }+\left(1-\chi \right)w^{\psi^{\prime }}$, while the update variable χ is typically small (χ≪1) to ensure the soft update of the target networks. The architectures of the actor and critic networks used in this work are adopted from the DDPG implementation in [38], including two hidden layers for the actor network and four hidden layers for the critic networks.

The computational complexity of the proposed TD3-based HEMS algorithm is primarily driven by the forward and backward passes of the neural networks during training. At each time step, the agent performs inference through the actor and twin critic networks, followed by gradient-based updates using a mini-batch of size *Y*. Since, in this work, we use fully connected networks with $N_{\psi }$, $N_{Q_{1}},N_{Q_{2}}$ parameters for the actor and the two critic networks respectively, the per-update complexity is $O(Y\left(N_{\psi }+2N_{Q}\right))$, assuming that the two critic networks are identical and $N_{Q_{1}}=N_{Q_{2}}=N_{Q}$. Since the TD3 framework employs twin critics and delayed policy updates, the critic networks are updated at every step, while the actor and target networks are updated every *d* steps, slightly reducing the overall computational burden compared to standard actor–critic methods with synchronous updates. Over a full training process consisting of *M* episodes and *N* time steps per episode, the total computational complexity scales as $O(MNY\left(N_{\psi }+2N_{Q}\right))$. The use of experience replay ensures that updates are independent of the trajectory length, while the clipping and noise injection operations introduce negligible overhead.

### 4.3. Federated Learning Algorithmic Process

**Figure 2:**
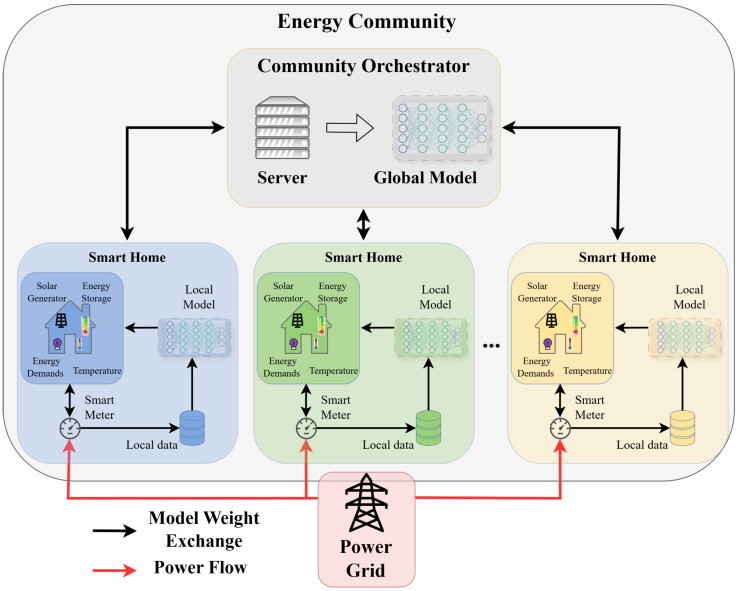
FL scheme among the smart homes inside an energy community, illustrating the fusion of ML model weights in the Community Orchestrator.

As aforementioned, the trained ML models of the individual TD3 agents are fused in a global ML model using FL. The architecture of the FL process is shown in Figure 2, where multiple smart homes that are inside the same energy community train their TD3 agents. Evidently, all community members (smart homes) have direct access to the power grid that covers their immediate energy deficit. It should be noted that the missing energy provided by the grid is typically the most expensive option, since it is the highest order of energy providers. Furthermore, a special logical entity is the Community Orchestrator (CO) that enables energy exchange between different communities through horizontal communication, and also serves as a message bus that conveys information between the various smart homes within the community. This information includes, for instance, energy plans or energy surpluses/deficits that are experienced by the community members. In the proposed scenario, we consider that the information that is conveyed from/to the community households to the CO are the updated neural network weights of the TD3 agents.

As an FL mechanism, pFedMe is adopted to deal with heterogeneous data originating from different smart homes. pFedMe enables a client-specific personalized control policy, using Moreau envelopes as client’s regularized loss, while maintaining coordination through a shared global model [52]. At each federated round, each smart home locally optimizes its TD3 policy using a proximal objective that penalizes deviation from the global policy. The server residing in the CO then aggregates the personalized models to update the parameters of the global model that are acknowledged back to the FL participants. In our implementation, the pFedMe proximal term is applied to both actor and critic parameters, yielding personalized policies and value functions while maintaining coordination through the global model. The global FL policy for all smart home clients is described by the vector $w=\{w^{\psi },w^{Q_{1}},w^{Q_{2}}\}$ that includes the global actor network and the twin critic networks of the TD3 model.

The FL algorithm is shown in Algorithm 2 and requires as input the smart home-specific data that are involved in Algorithm 1 from all the FL participants. After the initialization of the global policy $w^{0}$, the algorithm is executed for f=1,2,…,F federated rounds. In each FL round, each smart home/FL client receives the global model $w^{f-1}$ and then performs model training based on the local dataset, i.e., executes Algorithm 1 and updates the local TD3 model, aiming to minimize a Moreau envelope of the client objectives, allowing for controlled personalization via the regularization coefficient μ:

(21) $$w\leftarrow \arg\min_{w}L_{\ell }\left(w_{\ell }^{\psi },w_{\ell }^{Q_{1}},w_{\ell }^{Q_{2}}\right)+\frac{\mu }{2}\left(∥w_{\ell }^{\psi }-w^{\psi }{∥}^{2}+∥w_{\ell }^{Q_{1}}-w^{Q_{1}}{∥}^{2}+{∥w_{\ell }^{Q_{2}}-w^{Q_{2}}∥}^{2}\right),$$

where ℓ=1,2,…,L are the number of clients in the FL scheme and $L_{\ell }\left(w_{\ell }^{\psi },w_{\ell }^{Q_{1}},w_{\ell }^{Q_{2}}\right)$ is the TD3 loss for the $\ell^{\mathrm{th}}$ client. Finally, the average of the actor and critic network parameters is utilized to calculate the global policy for this federated round. In this context, each smart home learns its own policy, while the global model acts as a soft regularizer and the parameter μ controls the personalization vs. federation trade-off. It is worth noting that the overall complexity in the federated setting increases linearly with the number of participating clients O(L), although communication costs are limited to periodic model aggregation. Overall, the proposed approach remains computationally tractable for real-time HEMS applications, as the dominant operations are matrix multiplications that can be efficiently accelerated on modern hardware.
**Algorithm 2** pFedMe for Federated Smart Home Control**Input:** 
Algorithm 1 and related data for all participants/smart homes.**Output:** 
Global policy $w=\{w^{\psi },w^{Q_{1}},w^{Q_{2}}\}$ that includes the global actor network and the twin critic networks of the TD3 model. 1:Random initialization of global policy $w^{0}$ 2:**for** each federated round f=1,…,F **do** 3:   **for** each client *ℓ* in parallel **do** 4:     Receive $w^{f-1}={\left\{w^{\psi },w^{Q_{1}},w^{Q_{2}}\right\}}^{f-1}$ 5:     Execute Algorithm 1 with loss calculated by Equation (21) 6:   **end for** 7:   $w^{f}\leftarrow \frac{1}{L}\sum_{\ell =1}^{L}\left\{w_{\ell }^{\psi },w_{\ell }^{Q_{1}},w_{\ell }^{Q_{2}}\right\}$ 8:**end for**

## 5. Results

### 5.1. Individual TD3 Model Training

This subsection details the training details of a single smart home TD3 HEMS agent, focusing on the stabilization hyperparameters and the environmental variables. The simulation results presented herein are based on real data obtained from an open household dataset. Specifically, the time series of energy consumption and generation by renewable sources originating from several households, as well as the ambient temperature from the region of Germany, can be retrieved online (https://data.open-power-system-data.org/household_data/ accessed on 27 February 2026), along with the time series of the energy market. The rest of the parameters that are required to model the dynamic smart home environment (e.g., thermal building dynamics) are tabulated in Table 2, according to typical range values found in the literature [7,38]. Without the loss of generality, the ESS maximum capacity is adjusted to 10 kWh, the maximum power provided to the HVAC system is 8 kW, the comfortable temperature bounds are between 19.5 and 22.5 °C, and η=−1 is used for the value of the thermal conversion efficiency [53,54]. It is worth mentioning that these environmental parameters can be, in principle, different for each smart home, necessitating the use of personalized FL in the training process. In addition, Table 2 tabulates the learning parameters of the TD3 model, as described in Algorithm 1, that have been fine-tuned via simulations.

**Figure 3:**
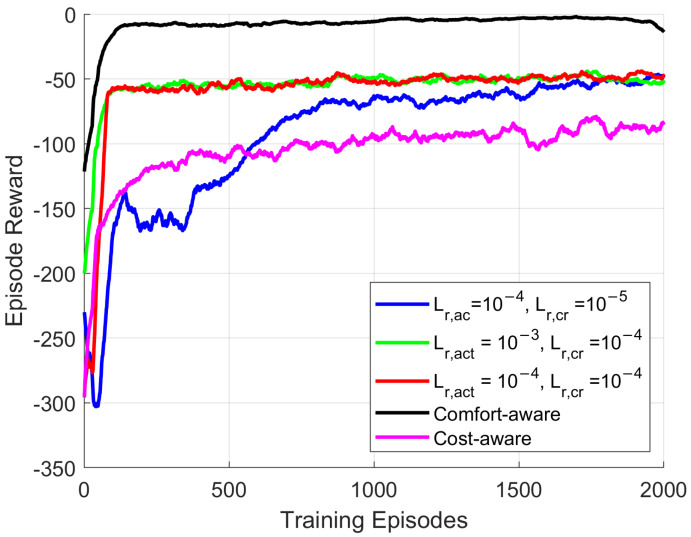
Learning curves for an individual TD3 HEMS agent for different values of the actor and critic learning rates, as well as different optimization objectives (cost-aware, balanced, comfort-aware).

The resulting fine-tuned learning curves are shown in Figure 3 for a training session consisting of M=2000 training episodes and N=24 inter-episode steps. In each episode, the TD3 agent interacts with the dynamic environment for 24 consecutive hours, aiming to learn the temporal dependencies of the associated parameters. The environment is reset in the next training episode at a random day and the TD3 agent interacts with the environment in a trial-and-error manner. According to the learning curves, the selected values are $L_{r,ac}=10^{-3}$ for the actor network and $L_{r,cr}=10^{-4}$ for the critic networks, whereas these results concern a balanced optimization scenario between the thermal comfort and the energy cost (ρ=10). Additional TD3 agents can be trained with different optimization objectives in the reward function for comfort-aware HEMS (ρ=0) and cost-aware HEMS (ρ=20), as depicted in Figure 3, depending on the specific requirements of the household resident. In the former case, the episode reward converges approximately to zero, since the TD3 agent learns to maintain the temperature within the requested limits regardless of the energy cost (HVAC system and ESS). On the other hand, the cost-aware HEMS agent prefers the temperature penalty in favor to the energy cost, converging to lower values. In principle, training the HEMS agent with ρ=10 is considered an adequate trade-off between the indoor comfort and the energy cost. It is worth mentioning that the performance of the TD3 agents are not compared to additional baseline methods herein, since previous studies have consistently reported that actor/critic techniques significantly increase the benefits for HEMS control compared to classical approaches (e.g., cognitive rule-based approaches, as well as DRL methods with discrete action space) in terms of energy cost and thermal comfort [7,38].

### 5.2. FL Training of TD3 Agents Within the Energy Community

Regarding the FL configuration of multiple smart home, Table 3 provides a qualitative comparison of the considered FL schemes in this work, highlighting their key characteristics in terms of personalization capability, robustness to data heterogeneity, and computational complexity. First, the FedAvg algorithm relies on simple model averaging and assumes homogeneous data distributions across clients, which limits its ability to adapt to the diverse operating conditions of different smart homes. Moreover, the FedProx approach partially addresses this issue by introducing a proximal regularization term that stabilizes local updates and improves convergence under heterogeneous data, yet it still enforces a single global model. In contrast, the proposed approach is based on the pFedMe method, explicitly enabling personalization by decoupling global and local objectives through Moreau envelope-based regularization, allowing each client to learn a model tailored to its specific environment while still benefiting from shared knowledge. This flexibility comes at the cost of increased computational complexity due to the additional optimization steps required for model personalization. Overall, pFedMe offers a more suitable framework for energy communities with heterogeneous participants, where individualized control policies are essential for achieving optimal performance.

**Figure 4:**
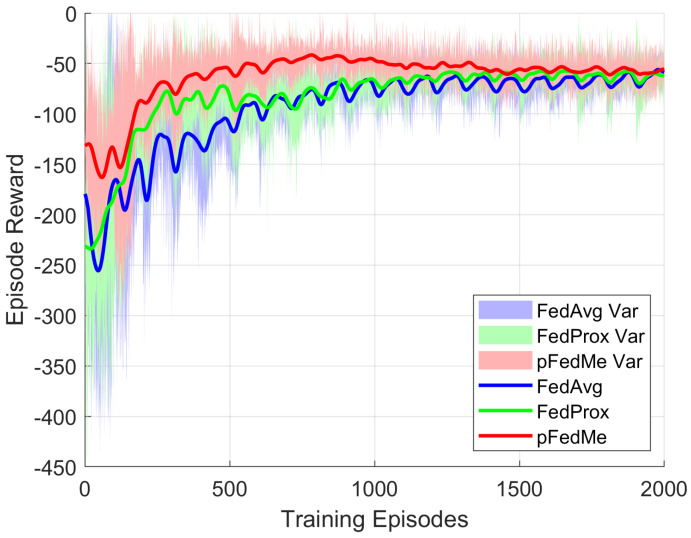
Training convergence for different FL algorithms, namely, FedAvg, FedProx and pFedMe for a configuration of three smart homes. The mean value and the variance of the episode reward between the FL participants are illustrated.

In this context, extending the training in the FL configuration of the energy community consisting of three smart homes, the learning curves for different FL algorithms are presented in Figure 4.

All the FL algorithms run for F=20 federated learning rounds, while keeping the total number of training episodes M=2000. In addition, the regularization coefficient is fine-tuned to the value of $\mu =10^{-3}$ for the pFedMe and $\mu =10^{-5}$ for the FedProx implementation and the three schemes have been trained for the balanced optimization scenario of ρ=10. Evidently, the pFedMe exhibits better learning behavior compared to the two baseline algorithms, since the mean episode reward converges after approximately 500 training episodes and remains close to the reward of the individual TD3 agent.

### 5.3. Evaluation Results of the FL Schemes

To evaluate the impact of the personalized FL algorithm and assess the performance of the trained HEMS agents residing in the smart homes, the individual TD3 models are tested with real data that were not utilized during the learning process. In this context, the agents are tested with data for 1 month that was not encountered during the training (namely, the ambient outdoor temperature, the renewable power generation, the energy consumption and the buying electricity price time series), while randomly initializing each smart home environment (ESS level, indoor temperature and time instance). It is worth noting that only the actor networks are used during the inference (evaluation) phase, since the output of the critic networks is required only during TD3 agent training to provide feedback related to the action selected by the actor network and update its policy.

**Figure 5:**
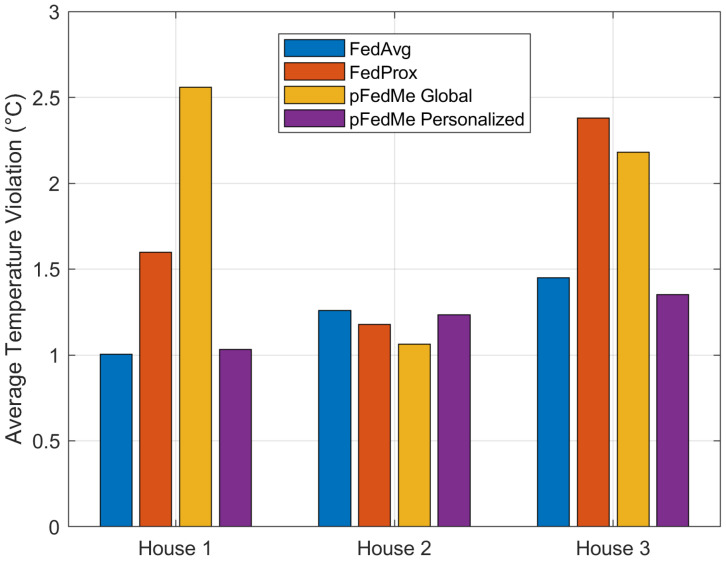
Average temperature violation for the different FL algorithms and the three smart homes inside the energy community.

The house-specific TD3 actor networks are inferred with the evaluation data in terms of two metrics: the average temperature violation in the inference period of 1 day (N=24) and the average daily energy cost. Regarding the temperature violation key performance indicator (KPI), the simulation results depicted in Figure 5 verify the efficiency of the pFedMe algorithm compared with the baselines of the FedAvg, FedProx and pFedMe global model. Notably, the latter baseline refers to the pFedMe model that has not conducted the final personalization steps during the FL process. Evidently, the pFedMe personalized TD3 agents achieve a temperature violation below 1.5 °C for all three houses, being marginally outperformed by FedAvg in house 1, as well as FedProx and pFedMe global in house 2.

In addition, Figure 5 illustrates the second KPI, i.e., the average daily energy cost for the different FL algorithms and the different households, where the pFedMe personalized models outperform all the baselines in all three houses. Specifically, the minimum reduction in the energy cost compared to FedAvg is approximately 37% for the first household, approximately 35% for the third smart home compared to Fedprox and less than 10% for the second household compared to the average daily energy cost provided by the pFedMe global model.

**Figure 6:**
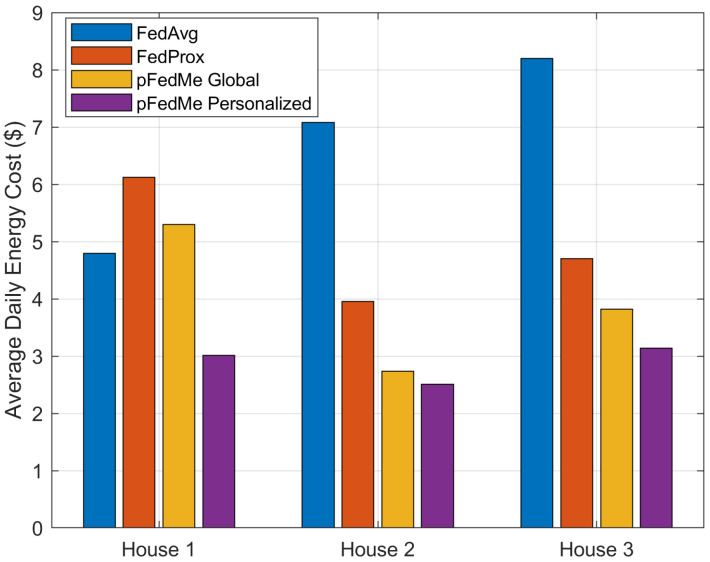
Average daily energy cost for the different FL algorithms and the three smart homes inside the energy community.

Overall, the superior performance of the personalized pFedMe scheme can be attributed to its ability to explicitly account for statistical heterogeneity across smart homes. In contrast to FedAvg, which enforces a single global model and may lead to sub-optimal decisions when local environments differ, and FedProx, which only constrains local updates through a proximal term, pFedMe introduces a Moreau envelope-based regularization that decouples global model learning from local personalization. This allows each agent to converge towards a solution that is both close to the global model and well-adapted to its local dynamics, such as distinct consumption patterns, thermal characteristics, and renewable generation profiles. As a result, the personalized policies learned by pFedMe achieve a better trade-off between energy cost and thermal comfort, leading to improved overall performance compared to non-personalized federated approaches. This behavior is also reflected in the reduced temperature violations and lower energy cost observed in Figure 5 and Figure 6.

## 6. Discussion and Conclusions

The present work aims to extend the intelligent HEMS agent from a single smart home to an energy community level, where the participating clients/end devices collaborate in an FL scheme to exchange intelligence distilled from the local data, environmental parameters and historical trial-and-error decisions. To this end, we present how the collaborative training of multiple TD3 agents can be conducted in a PFDRL framework, using the Community Orchestrator as the aggregation entity that performs the fusion of the global model. We differentiate from previous studies by proposing a personalized FL algorithm that uses Moreau envelopes (pFedMe), decoupling the personalized model optimization from the global model learning. The introduced pFedMe method manages to maintain an optimal trade-off between the generalization capabilities offered by the typical FL mechanism and personalized model training that may result in overfitting. In other words, the intelligent agent of an individual smart home trained in the presented scheme copes with heterogeneous data that may be encountered due to the FL process and at the same time provides improved performance for the local environment. The simulation results clearly illustrate the performance capabilities of the TD3 personalized agents trained in the pFedMe scheme compared to the baselines of the FedAvg, Fedprox and pFedMe global model outputs. Specifically, the pFedMe scheme achieves a maximum temperature violation below 1.5 °C for the three houses in the FL scheme, exhibiting similar results with the FedAvg and FedProx frameworks. In addition, the pFedMe personalized agents outperform the baseline FL schemes in all three houses regarding the average daily energy cost, demonstrating an improvement of at least 37%, 35% and 10% for the three residential houses, respectively.

Regarding future work, we aim to include different thermal comfort variables in the modeling of the smart home (e.g., humidity), complementing also the control actions of the TD3 agent. Moreover, we plan to extend this approach in energy communities that also exchange directly locally produced energy to achieve a zero-sum energy footprint. Finally, other than including smart homes in the FL scheme, we aim to incorporate different types of participating clients/end devices (e.g., EV chargers) in the FL scheme that may act as prosumers in the energy community.

## Figures and Tables

**Table 1:** Mathematical symbols.

Symbol	Description	Symbol	Description
$\omega_{t}$	Smart home energy demands [kWh]	$T_{t}^{out}$	Ambient temperature [°C]
*A*	Thermal conductivity [kW/°f]	ϵ	Inertia factor
η	HVAC performance coefficient	$T_{t}^{in}$	Indoor temperature [°C]
$e_{t}$	HVAC provided power [kW]	$e^{max}$	Peak HVAC power [kW]
$T^{min}$	Lower bound of $T_{t}^{in}$ [°C]	$T^{max}$	Upper bound of $T_{t}^{in}$ [°C]
$C_{t}$	ESS charge level [kWh]	$\delta_{\sigma }$	Charging coefficient
$\sigma_{t}$	Charging rate [kW]	$\lambda_{t}$	Discharging rate [kW]
$\delta_{\lambda }$	Discharging coefficient	$C^{min}$	Lower bound of $C_{t}$ [kWh]
$C^{max}$	Upper bound of $C_{t}$ [kWh]	$\sigma^{max}$	Upper bound of $\sigma_{t}$ [kW]
$\lambda^{max}$	Upper bound of $\lambda_{t}$ [kW]	$p_{t}$	Power from renewables [kW]
$\gamma_{t}$	Power from/to the grid [kW]	$\kappa_{t}$	Buying electricity price [$/kW]
$\beta_{t}$	Selling electricity price [$/kW]	ξ	Price scaling factor
υ	ESS depreciation coefficient [$/kW]	$s_{t}$	Smart home state
$\alpha_{t}$	TD3 agent action	${\hat{e}}_{t}$	Scaled HVAC input power
${\hat{\zeta }}_{t}$	Scaled ESS charging/discharging variable	$r_{t}$	Reward function
ρ	Trade-off coefficient	$Q(s_{t},\alpha_{t})$	Action value function
R	Replay memory	$w^{Q}$	Critic network weights
$w^{\psi }$	Actor network weights	*M*	Training episodes
*N*	Within-episode time slots	ν	Action exploration noise of deviation $\sigma_{n}^{2}$
*Y*	Mini-batch size	$\nu_{c}$	Clipping limit of exploration noise
θ	Discount factor	$N_{\psi }$	Dimensions of the actor networks
$N_{Q_{1}}$	Dimensions of the critic networks	w	Global FL policy
μ	Regularization coefficient for the personalization	*L*	Number of clients in the FL scheme
L	TD3 loss for pFedMe	*F*	Number of federated rounds

**Table 2:** Simulation parameters of the smart home environment and the TD3 learning algorithm.

Environment Parameters	Value	Learning Parameters	Value
ϵ	0.7	$\sigma_{n}^{2}$	0.2
η	−1	*M*	2000
*A* (kW/°f)	0.14	*N*	24
$e^{max}$ (kW)	8	*K*	35,000
$T^{max}$ (°C)	22.5	*Y*	32
$T^{min}$ (°C)	19.5	*c*	0.5
$C^{max}$ (kWh)	10	θ	0.99
$C^{min}$ (kWh)	0.6	χ	$10^{-3}$
$\delta_{\sigma },\delta_{\lambda }$	0.95	$N_{\psi }$	(300, 600)
$\sigma^{max},\lambda^{max}$ (kW)	3	$N_{Q_{1}},N_{Q_{2}}$	(300, 600, 600, 600)
ξ	1.1	*F*	20
υ ($/kW)	1	μ	$10^{-3}$

**Table 3:** Qualitative comparison of FL schemes.

Property	FedAvg	FedProx	pFedMe
Global model sharing	✓	✓	✓
Personalized models	✗	✗	✓
Handles data heterogeneity	Low	Medium	High
Regularization to global model	✗	✓(proximal term)	✓(Moreau envelope)
Decoupling global/local objectives	✗	Partial	✓
Convergence stability	Medium	High	High
Adaptation to local preferences	Low	Medium	High
Communication efficiency	High	High	Medium

## Data Availability

The following open-source datasets were used in this work: (1) Data related to the energy management of residential households can be found here: https://data.open-power-system-data.org/household_data/ accessed on 27 February 2026. (2) Data related to the energy market can be found here: https://data.nordpoolgroup.com/auction/day-ahead/prices?deliveryDate=latest&currency=EUR&aggregation=Hourly&deliveryAreas=AT accessed on 27 February 2026.
